# Agricultural Pesticide Exposure and Antimicrobial Resistance in *Escherichia coli* Across 28 European Countries: A Panel and Spatial Data Analysis

**DOI:** 10.3390/microorganisms14092018

**Published:** 2026-09-11

**Authors:** Meryem Toprak Tuncer, Tuba Bayir, Ahmet Atessahin

**Affiliations:** 1 Department of Pharmacology and Toxicology, Faculty of Veterinary Medicine, Fırat University, Elazig 23200, Türkiye; m.toprak@firat.edu.tr; 2 Department of Biometry, Faculty of Veterinary Medicine, Fırat University, Elazig 23200, Türkiye; tbayir@firat.edu.tr

**Keywords:** antimicrobial resistance, pesticide use, antibiotic consumption, panel data analysis, spatial autocorrelation

## Abstract

Antimicrobial resistance (AMR) is a growing global health threat, and agricultural pesticide exposure has been proposed as an environmental driver of resistance alongside antibiotic consumption. However, long-term, multi-country evidence linking pesticide use to AMR in *Escherichia coli* remains limited. Panel data analysis was applied to data from 28 European countries between 2013 and 2023 to examine lagged associations between agricultural pesticide use and *E. coli* resistance to fluoroquinolones, third-generation cephalosporins, aminoglycosides, and aminopenicillins, alongside spatial analysis of resistance distribution. Country-level panel data on *E. coli* resistance, antibiotic consumption, and pesticide use per cultivated area were compiled for 28 European countries, except for the aminopenicillin resistance model, for which Sweden was excluded owing to insufficient longitudinal data, yielding a 27-country panel for that indicator. Four random-effects generalized least squares (GLS) panel regression models were constructed for each resistance indicator, incorporating same-year and one-, two-, and three-year lagged pesticide use, adjusted for the corresponding antibiotic consumption. Global Moran’s I and Local Indicators of Spatial Association (LISA) analyses assessed spatial autocorrelation and clustering of resistance across countries. Pesticide use was significantly and positively associated only with fluoroquinolone resistance at a three-year lag; no significant associations were found for third-generation cephalosporin, aminoglycoside, or aminopenicillin resistance at any lag. Antibiotic consumption was consistently and positively associated with resistance across all models. All four resistance indicators showed statistically significant positive spatial autocorrelation, with persistent high-resistance clusters in Southeast Europe and low-resistance clusters in Northern Europe. Antibiotic consumption remains the dominant determinant of *E. coli* resistance, whereas pesticide use shows only a delayed, class-specific association restricted to fluoroquinolone resistance.

## 1. Introduction

Antimicrobial resistance (AMR) has become one of the most significant global health challenges for human and animal health. Worldwide, approximately 1.27 million deaths have been caused by infections resulting from resistant bacteria, while 4.95 million deaths have been attributed to these infections [1,2]. This significant problem means that the therapeutic successes achieved in modern medicine are at risk. Misuse of antibiotics in human, animal, and plant production is recognized as the primary driver of AMR emergence and spread. For this reason, the “One Health” approach, which addresses human, animal, and environmental health collectively, is increasingly coming to the forefront in the fight against AMR. One Health emphasizes that the health of humans, animals, and ecosystems is interconnected, highlighting that resistant organisms can spread rapidly through healthcare, livestock, food, and the environment and thus, a solution requires cross-sectoral collaboration [3]. In this context, establishing effective monitoring systems, tracking resistance trends across different sectors, and providing early warning and preventive measures are considered crucial.

*Escherichia coli* (*E. coli*) is one of the indicator bacteria frequently used in AMR surveillance to monitor the prevalence of antibiotic resistance and its changes over time. Found as a commensal in the intestines of both humans and animals, this bacterium is known to be a leading cause of common clinical diseases such as gastrointestinal infections, urinary tract infections, and sepsis through its pathogenic strains. The genomic plasticity of *E. coli* and its ability to colonize diverse environments make it an ideal model organism for studying AMR mechanisms [4]. For this reason, *E. coli* has been selected as the standard indicator organism in human, food, and environmental samples in the official resistance surveillance programs of many countries. Certain classes of antibiotics monitored in *E. coli* are considered particularly important from a public health perspective. For example, fluoroquinolones and third-generation cephalosporins are classified as “highest-priority, critically important antimicrobials” in human medicine [5]. Consequently, the development of resistance to these classes can lead to treatment failures. Similarly, while aminoglycosides (particularly gentamicin) are a treatment option for many serious Gram-negative infections, resistance to this group can reduce the effectiveness of combination therapies in particular [6]. On the other hand, aminopenicillins have been used for many years as the first-line treatment against enteric bacteria such as *E. coli* due to their safety margins and have shown the highest resistance rates in *E. coli* strains in many countries [7,8]. Consequently, resistance trends in these four classes of antibiotics are being closely monitored in terms of the management of *E. coli*-associated infections and public health risks.

Excessive or inappropriate use of antibiotics is not the only factor contributing to the development of AMR. Various chemicals and heavy metals that accumulate in the environment can exert selective pressure on bacteria even in the absence of antibiotics [9]. For example, chlorine compounds and quaternary ammonium compounds used as disinfectants, or heavy metals frequently applied in agriculture, can indirectly trigger the development of antibiotic resistance in microorganisms [10], and a phenomenon known as co-selection plays a role in this process. It is believed that the presence of non-antibiotic agents may serve as a target for resistant bacteria and antibiotic resistance genes (ARGs), either through the co-carriage of ARGs and other resistance genes on the same mobile genetic elements (co-resistance) or through the development of cross-resistance, where a single mechanism confers resistance to multiple substances [11]. Consequently, environments with high concentrations of non-antibiotic agents can influence the spread of resistance genes. In recent years, research has increasingly focused on the impact of agricultural activities and environmental exposure on AMR. In addition to intensive livestock farming practices where antibiotic use is high, it is believed that environmental selection pressure in regions with intensive agriculture may contribute to the development of resistance. Among these environmental factors, pesticide use is of particular concern. Pesticides are chemicals widely used in large quantities in modern agriculture to maintain crop yields. Along with the increase in agricultural production, it has been reported that the intensity of pesticide use worldwide has reached levels of billions of kilograms in recent years [12,13,14]. Pesticide applications expose non-target environmental microorganisms to these substances for extended periods and high concentrations. However, the effects of agricultural pesticides on microbial ecosystems and resistance have long been neglected in risk assessments.

The European continent serves as a prime example of regional variations in the burden of AMR. Resistance rates for *E. coli* and similar common pathogens in European Union (EU) countries can vary by country, with higher resistance rates generally reported in southern and eastern European countries [15]. While this heterogeneity stems largely from country-specific antibiotic use policies, healthcare infrastructure, and hygiene conditions, it is believed that environmental and agricultural factors may also contribute to these differences between countries. In recent years, One Health-focused policies have been adopted in Europe to combat AMR, and member states have been advised to develop national action plans that integrate the human, animal, and environmental health sectors [16]. However, despite existing policy frameworks, there appears to be a limited amount of long-term, comparable data on the impact of agricultural chemicals and environmental exposure on the AMR problem at the national level. Existing studies either provide mechanism-focused evidence based on specific samples at the laboratory and field levels or focus on antibiotic use in human and veterinary medicine. Moreover, European countries are not spatially independent with respect to agricultural practices and environmental exposures. Pesticide use and environmental pesticide burdens show marked geographical heterogeneity across Europe, while pesticide residues are widely detected in European agricultural soils and may persist and disperse beyond their areas of application through interconnected environmental pathways [17,18]. At the same time, AMR patterns may also exhibit geographical dependence, as neighboring countries can share environmental, socioeconomic, agricultural, and cross-border transmission dynamics. Therefore, examining the spatial distribution of AMR may reveal geographical clustering and local high or low resistance patterns that cannot be identified through country-level temporal analyses alone. In this context, spatial analyses of AMR can provide a complementary perspective to panel regression models assessing the association between pesticide use and AMR [19]. With the development of open-access data sources, it has become possible to conduct large-scale analyses spanning different countries and years. However, there are few studies in the literature that comparatively evaluate the relationship between pesticide exposure and AMR over the long term and across multiple countries, and there remains a significant knowledge gap in this area [20].

The aim of this study is to address this gap by examining the relationship between pesticide use per cultivated area and reported resistance rates in *E. coli* to fluoroquinolones, aminoglycosides, third-generation cephalosporins, and aminopenicillins across 28 European countries between 2013 and 2023, using 11 years of up-to-date data from international open-source indicators. In doing so, the study seeks to generate comparable, long-term evidence on the link between pesticide pressure and AMR, and to offer a new perspective for AMR policy, surveillance, and future resistance-management strategies within a One Health framework.

## 2. Results

### 2.1. Results of Panel Data Regression Analysis

This section presents the results of the four random-effects GLS panel regression models assessing the association between pesticide use (same-year and one-, two-, and three-year lags) and *E. coli* resistance for each antibiotic class, adjusted for the corresponding antibiotic consumption; full regression estimates, including 95% confidence intervals, are shown in Table 1.

In the models developed for fluoroquinolone resistance, no statistically significant relationship was found between pesticide consumption in the same year and resistance. Similarly, no statistically significant relationship was found between one-year and two-year lagged pesticide consumption. In contrast, three-year lagged pesticide consumption showed a statistically significant positive association with fluoroquinolone resistance. On the other hand, fluoroquinolone consumption maintained a positive and statistically significant association with resistance in all models. When evaluating third-generation cephalosporin resistance, no statistically significant association with resistance was detected in any of the pesticide consumption or lagged models for the same year.

In contrast, third-generation cephalosporin consumption showed a statistically significant positive association with resistance in all models. For aminoglycoside resistance, no statistically significant association was found in any of the lagged models for pesticide consumption from the same year. In contrast, aminoglycoside consumption showed a positive and statistically significant association with resistance in the same-year, one-year, and two-year lagged models. In the three-year lagged model, the relationship between aminoglycoside consumption and resistance was found to be at the threshold of statistical significance. Similarly, no statistically significant relationship was found between pesticide consumption and aminopenicillin resistance in any of the same-year or lagged models. In contrast, aminopenicillin consumption showed a positive and statistically significant relationship with aminopenicillin resistance in all models.

Overall, pesticide consumption showed a significant positive association with fluoroquinolone resistance in the three-year lagged model but did not show a significant association with third-generation cephalosporin, aminoglycoside, or aminopenicillin resistance. In contrast, consumption variables for the relevant antibiotic classes showed a positive and statistically significant association with AMR in the vast majority of the models examined.

### 2.2. Results of the Spatial Analysis

#### 2.2.1. Spatial Distribution of Fluoroquinolone Resistance

**Figure 1:**
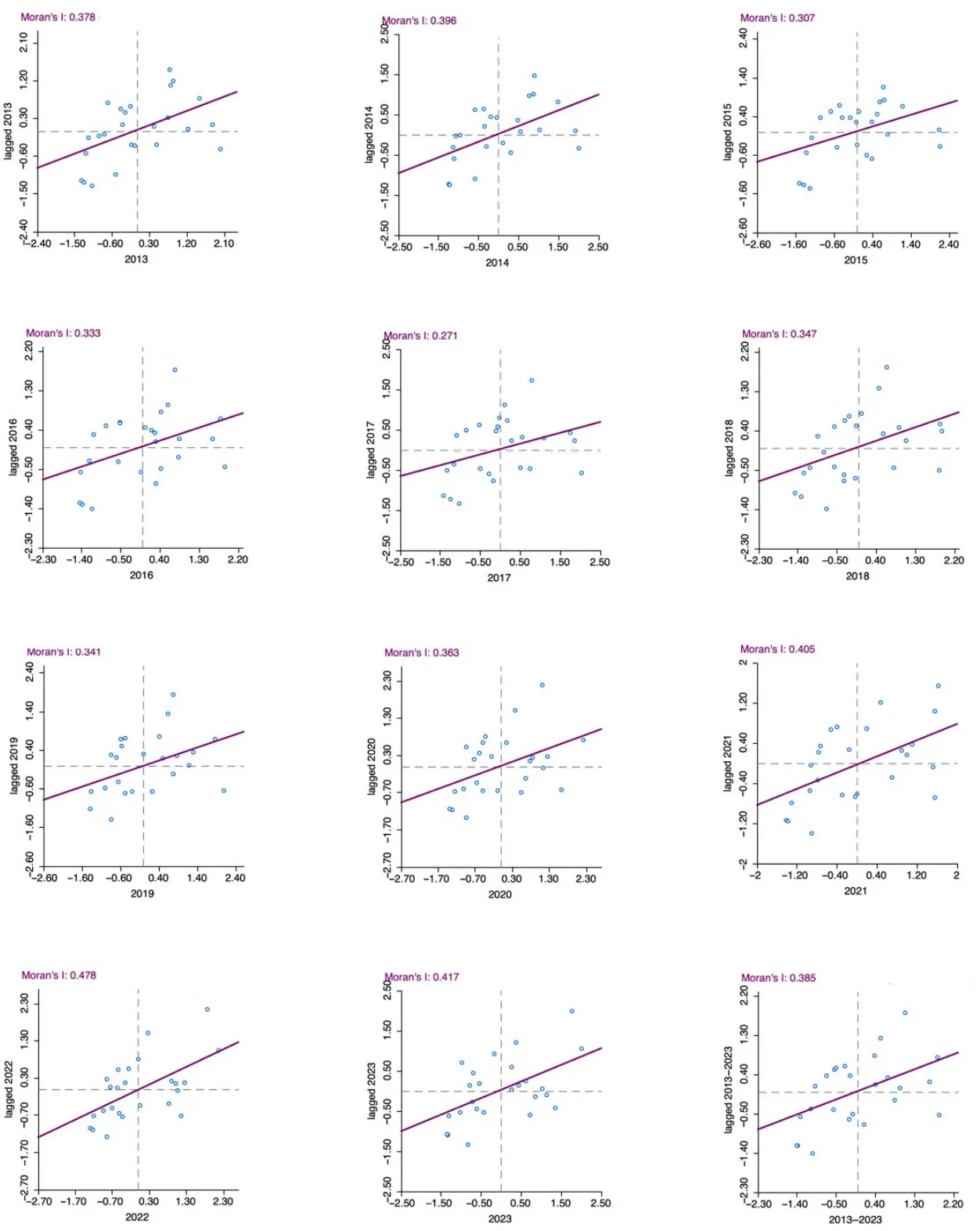
Moran’s I scatter plots illustrating the spatial autocorrelation of fluoroquinolone resistance across European countries from 2013 to 2023 and for the overall study period. Blue circles represent countries; the x-axis shows standardized resistance values, the y-axis shows spatially lagged values, dashed lines indicate zero reference values, and the purple regression line represents Moran’s I.

The global Moran’s I analysis revealed that fluoroquinolone resistance exhibited statistically significant positive spatial autocorrelation among European countries from 2013 to 2023. The Moran’s I coefficient ranged from 0.271 to 0.478 during the study period and was found to be positive and statistically significant in all years (*p* < 0.05). The lowest spatial autocorrelation was observed in 2017 (I = 0.271), and the highest in 2022 (I = 0.478). An analysis using 11-year average resistance data also revealed a significant positive spatial autocorrelation (I = 0.385; *p* = 0.008) (Figure 1).

**Figure 2:**
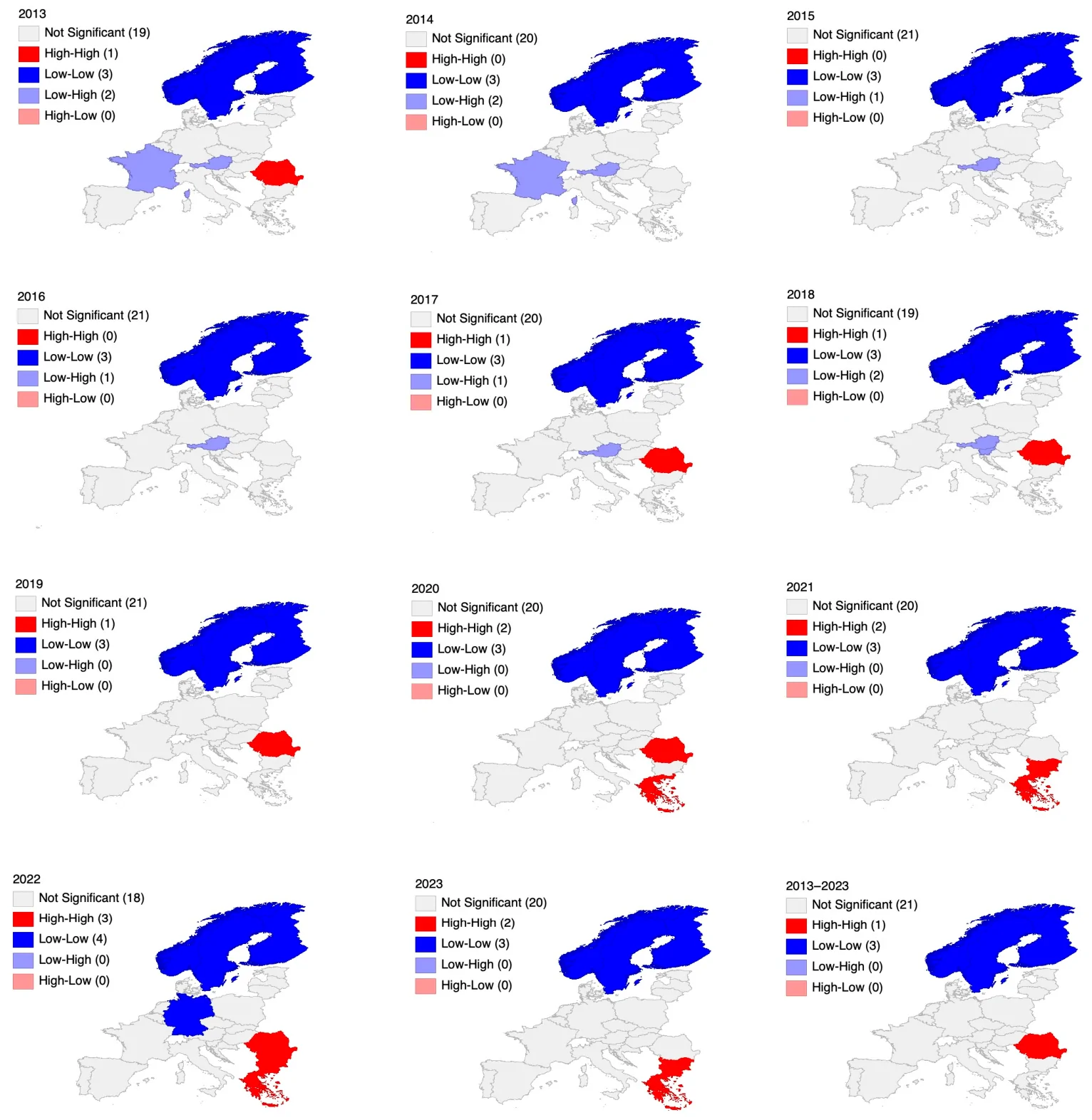
LISA cluster maps showing the spatial distribution of fluoroquinolone resistance across European countries from 2013 to 2023 and for the overall study period.

The Local Spatial Autocorrelation (LISA) analysis revealed that high-resistance clusters persisted over the years in certain regions. During the early years of the study (2013–2019), High–High clusters were observed only in Romania, but in 2020, Greece joined Romania in the high-resistance cluster. In 2021, both Greece and Bulgaria were observed as high-resistance clusters. In 2022, the number of High–High clusters rose to three, with Romania rejoining Greece and Bulgaria. In 2023, high-resistance clusters persisted in Bulgaria and Greece. In an analysis using average values for the entire study period, the High–High cluster was identified only in Romania. In contrast, Low–Low clusters exhibited a stable distribution across Northern Europe throughout all years. Norway, Sweden, and Finland were part of the low-resistance clusters during the study period, and Germany was added to this cluster in 2022. Low–High clusters, on the other hand, were observed only in a limited number of countries during certain years; France and Austria were in this group in 2013 and 2014, while Austria was the primary country in this group between 2015 and 2018. No significant country was identified as belonging to the High–Low cluster during the period under review (Figure 2).

#### 2.2.2. Spatial Distribution of Third-Generation Cephalosporin Resistance

**Figure 3:**
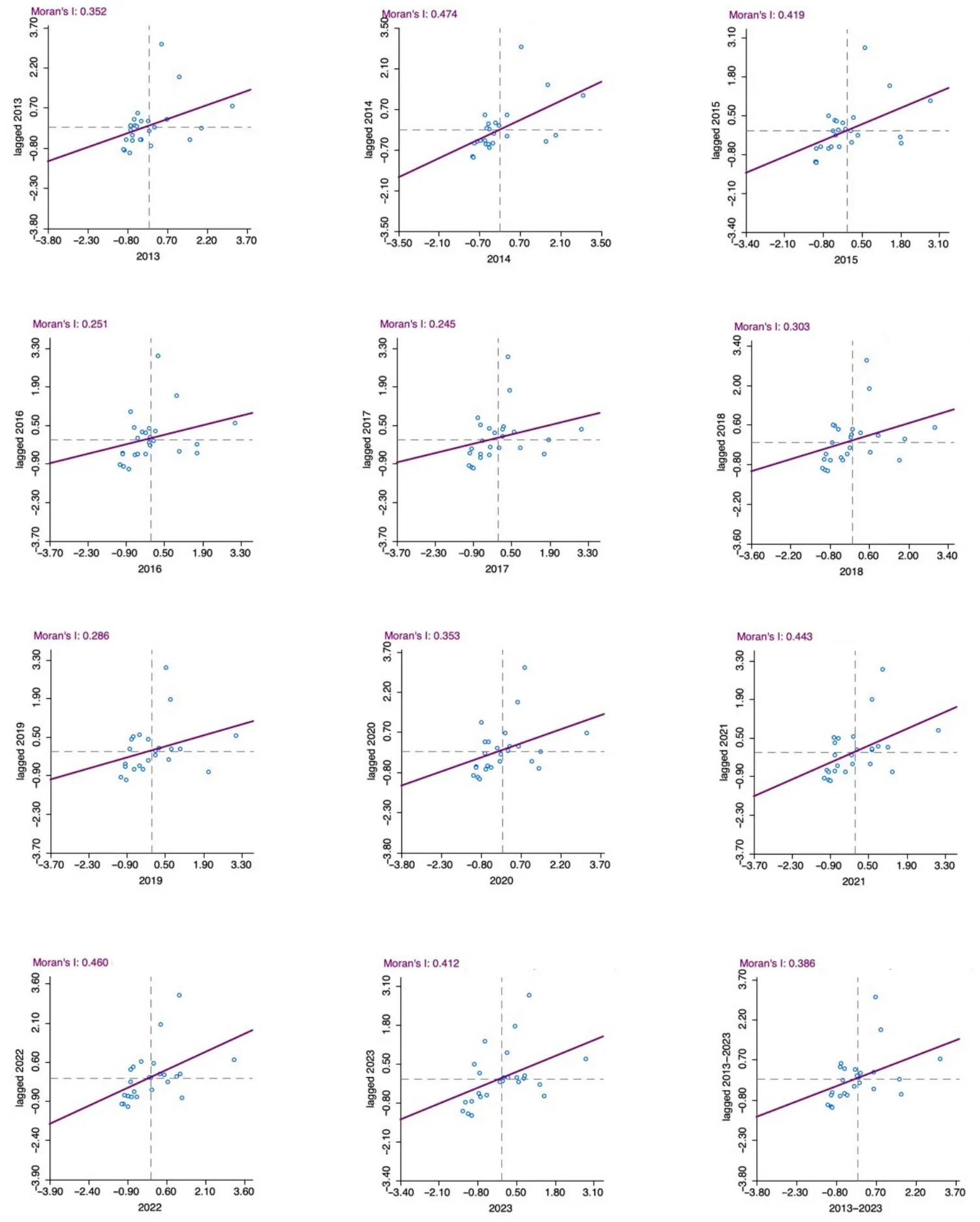
Moran’s I scatter plots illustrating the spatial autocorrelation of third-generation cephalosporin resistance across European countries from 2013 to 2023 and for the overall study period. Blue circles represent countries; the x-axis shows standardized resistance values, the y-axis shows spatially lagged values, dashed lines indicate zero reference values, and the purple regression line represents Moran’s I.

The global Moran’s I analysis revealed that third-generation cephalosporin resistance exhibited positive spatial autocorrelation among European countries for most of the study period. While the Moran’s I coefficient ranged from 0.245 to 0.474, spatial autocorrelation was found to be statistically significant in all years except 2016 and 2017 (*p* < 0.05). An analysis using average resistance data for the entire study period also revealed significant positive spatial autocorrelation (Moran’s I = 0.386; *p* = 0.009). The presence of a positively sloped regression line in Moran’s scatter plots for all years indicates that third-generation cephalosporin resistance tends to cluster spatially across Europe (Figure 3).

**Figure 4:**
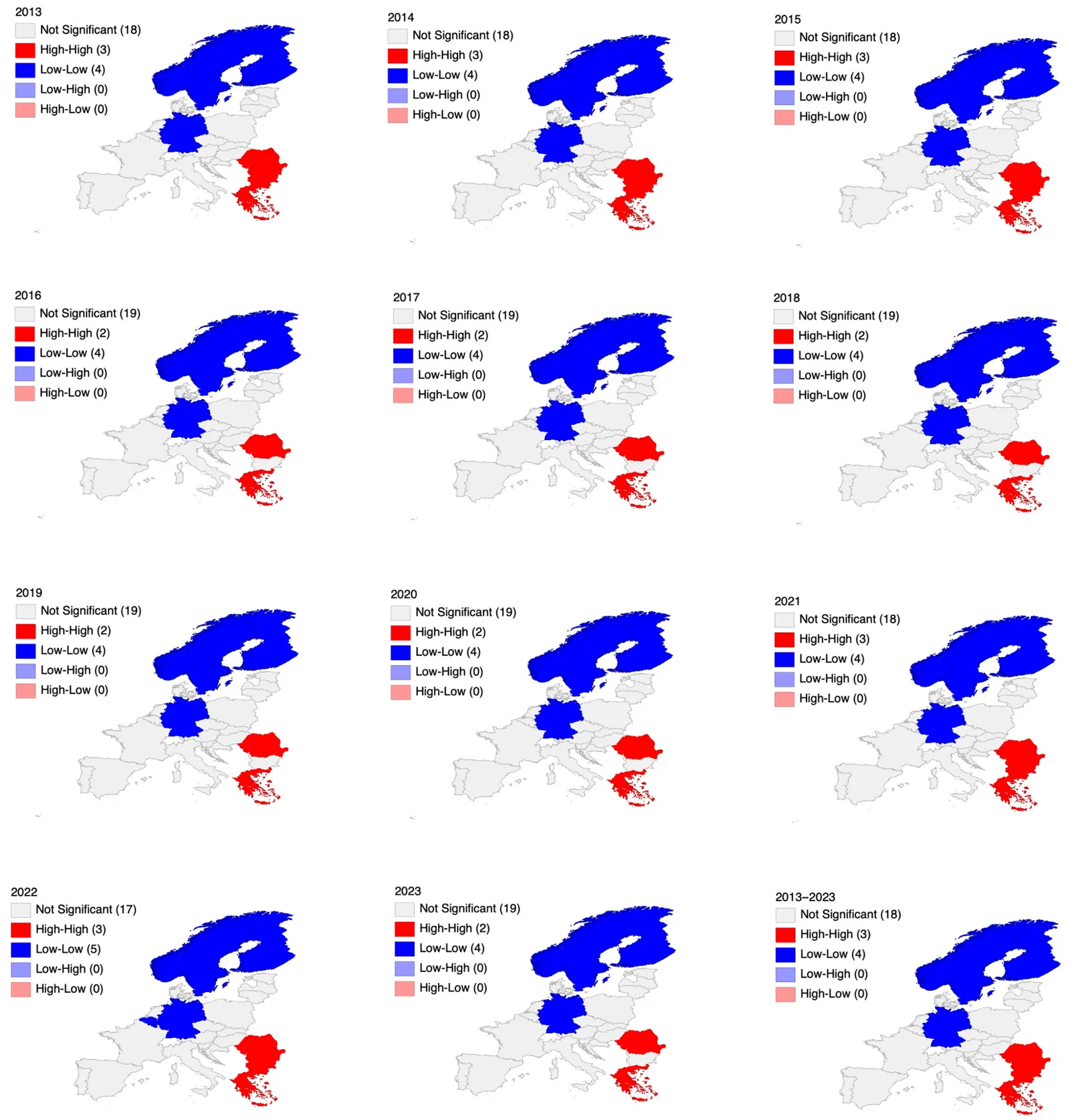
LISA cluster maps showing the spatial distribution of third-generation cephalosporin resistance across European countries from 2013 to 2023 and for the overall study period.

The LISA analysis showed that high-resistance clusters were concentrated primarily in Southeast Europe throughout the study period. While Romania, Bulgaria, and Greece formed significant High–High clusters in most years, high-resistance clusters were limited to Romania and Greece during the 2016–2020 period following Bulgaria’s departure from the cluster. In contrast, Norway, Sweden, Finland, and Germany formed stable Low–Low clusters throughout the study period, with Belgium joining this cluster in 2022. No significant Low–High or High–Low spatial outlier clusters were identified during the period under review (Figure 4).

#### 2.2.3. Spatial Distribution of Aminoglycoside Resistance

**Figure 5:**
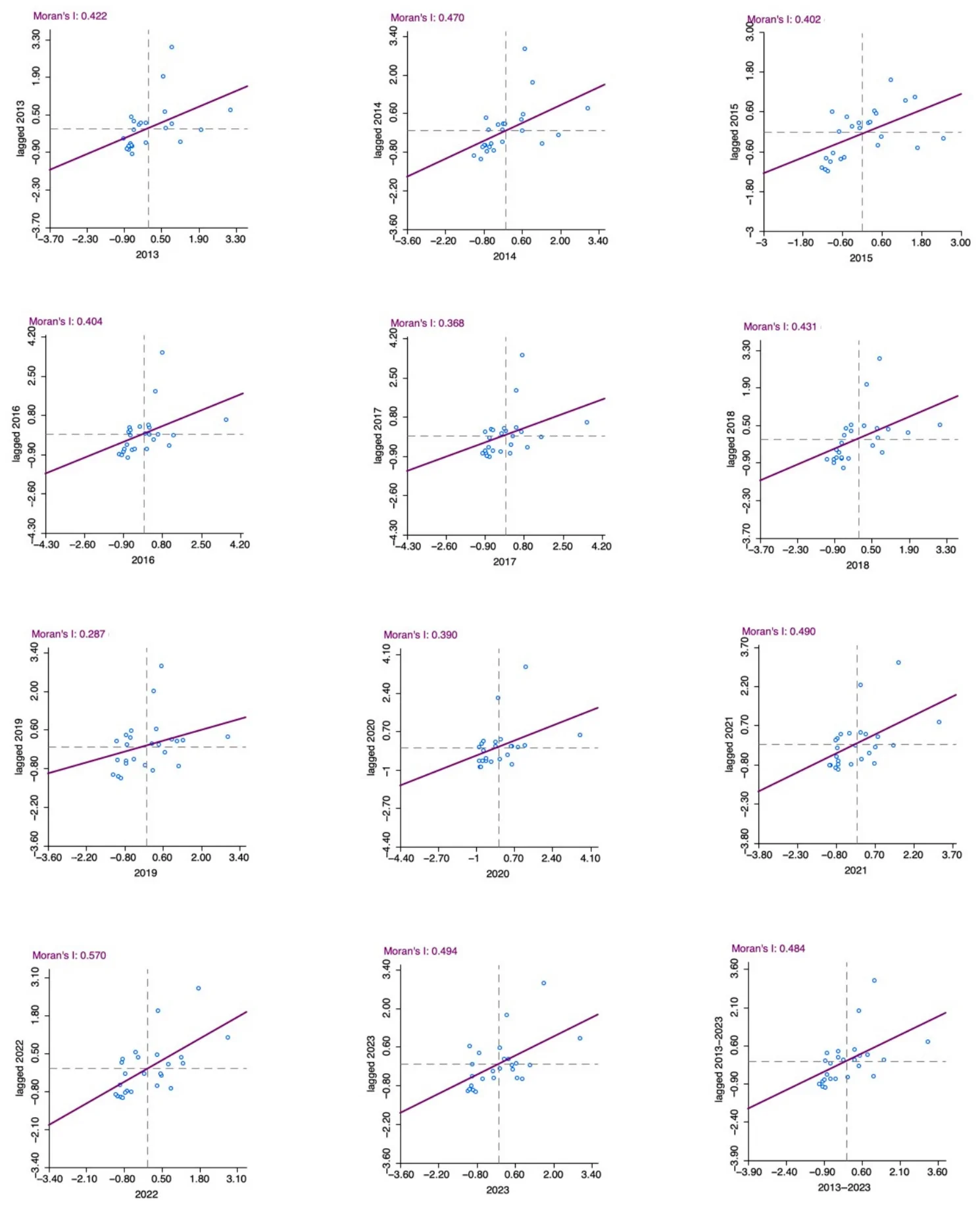
Moran’s I scatter plots illustrating the spatial autocorrelation of aminoglycoside resistance across European countries from 2013 to 2023 and for the overall study period. Blue circles represent countries; the x-axis shows standardized resistance values, the y-axis shows spatially lagged values, dashed lines indicate zero reference values, and the purple regression line represents Moran’s I.

A global Moran’s I analysis revealed that aminoglycoside resistance exhibited statistically significant positive spatial autocorrelation among European countries from 2013 to 2023. The Moran’s I coefficient ranged from 0.287 to 0.570 during the study period and was found to be positive and statistically significant in all years (*p* < 0.05). The lowest spatial autocorrelation was observed in 2019 (I = 0.287), and the highest in 2022 (I = 0.570). An analysis using average resistance data for the entire study period also revealed significant positive spatial autocorrelation (Moran’s I = 0.484; *p* = 0.003). The presence of a positively sloped regression line in Moran’s scatter plots for all years indicated that aminoglycoside resistance exhibited a distinct spatial clustering trend across Europe (Figure 5).

**Figure 6:**
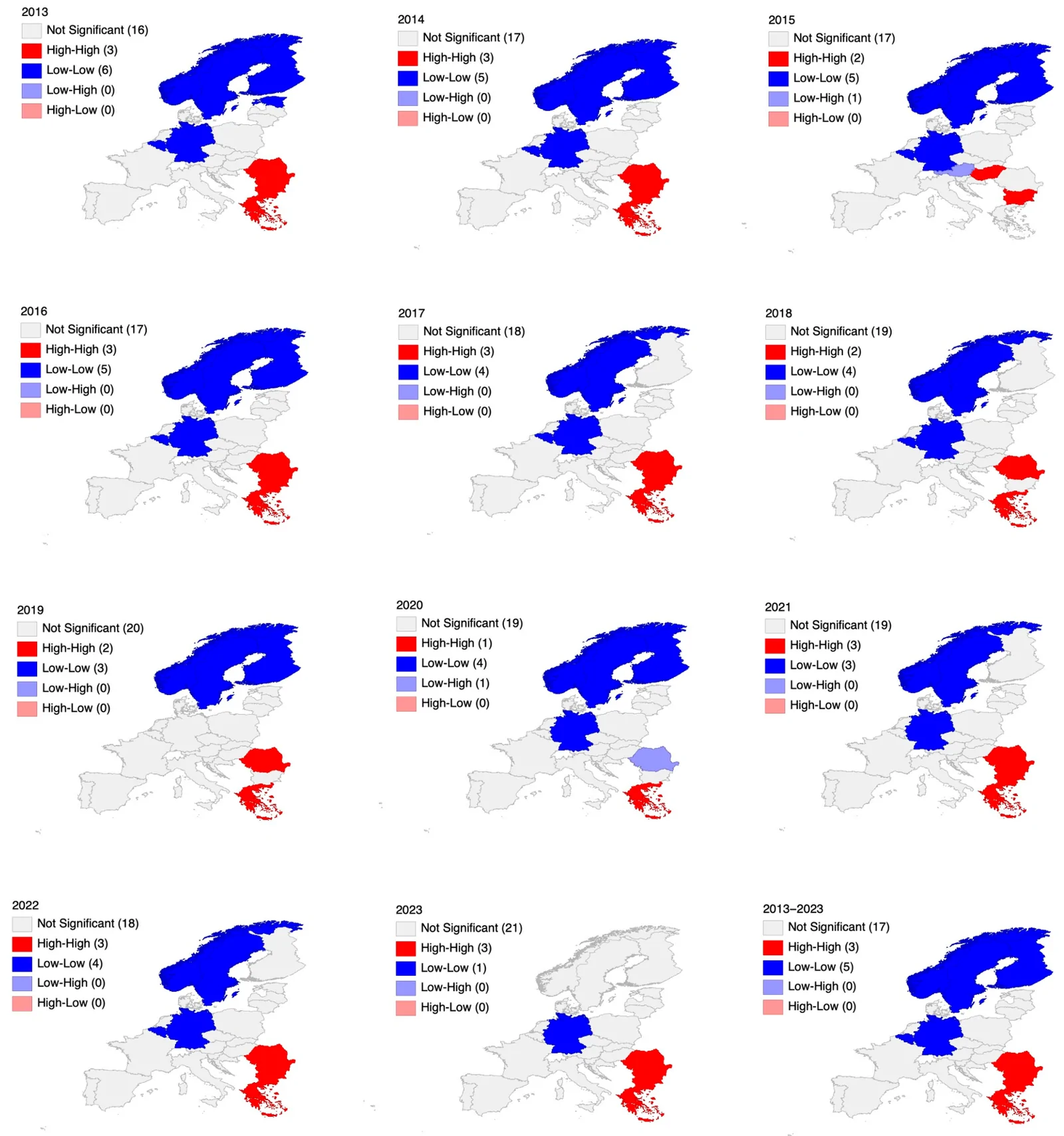
LISA cluster maps showing the spatial distribution of aminoglycoside resistance across European countries from 2013 to 2023 and for the overall study period.

According to the LISA analysis, high-resistance clusters were concentrated primarily in Southeast Europe throughout the study period. Romania, Bulgaria, and Greece formed significant High–High clusters in 2013, 2014, 2016, 2017, 2021, 2022, 2023, and in the overall analysis of the study period. In 2015, 2018, and 2019, with Bulgaria’s exclusion from the cluster, high-resistance clusters were limited to Romania and Greece; in 2020, only Greece was included in the High–High cluster. Low-resistance clusters were predominantly observed in Northern Europe throughout the study period. Norway, Sweden, Finland, Germany, and Belgium were part of the low-resistance clusters in many years. During the study period, the Low–High spatial outlier was observed only in Austria in 2015 and in Romania in 2020; the High–Low cluster was not observed in any year (Figure 6).

#### 2.2.4. Spatial Distribution of Aminopenicillin Resistance

**Figure 7:**
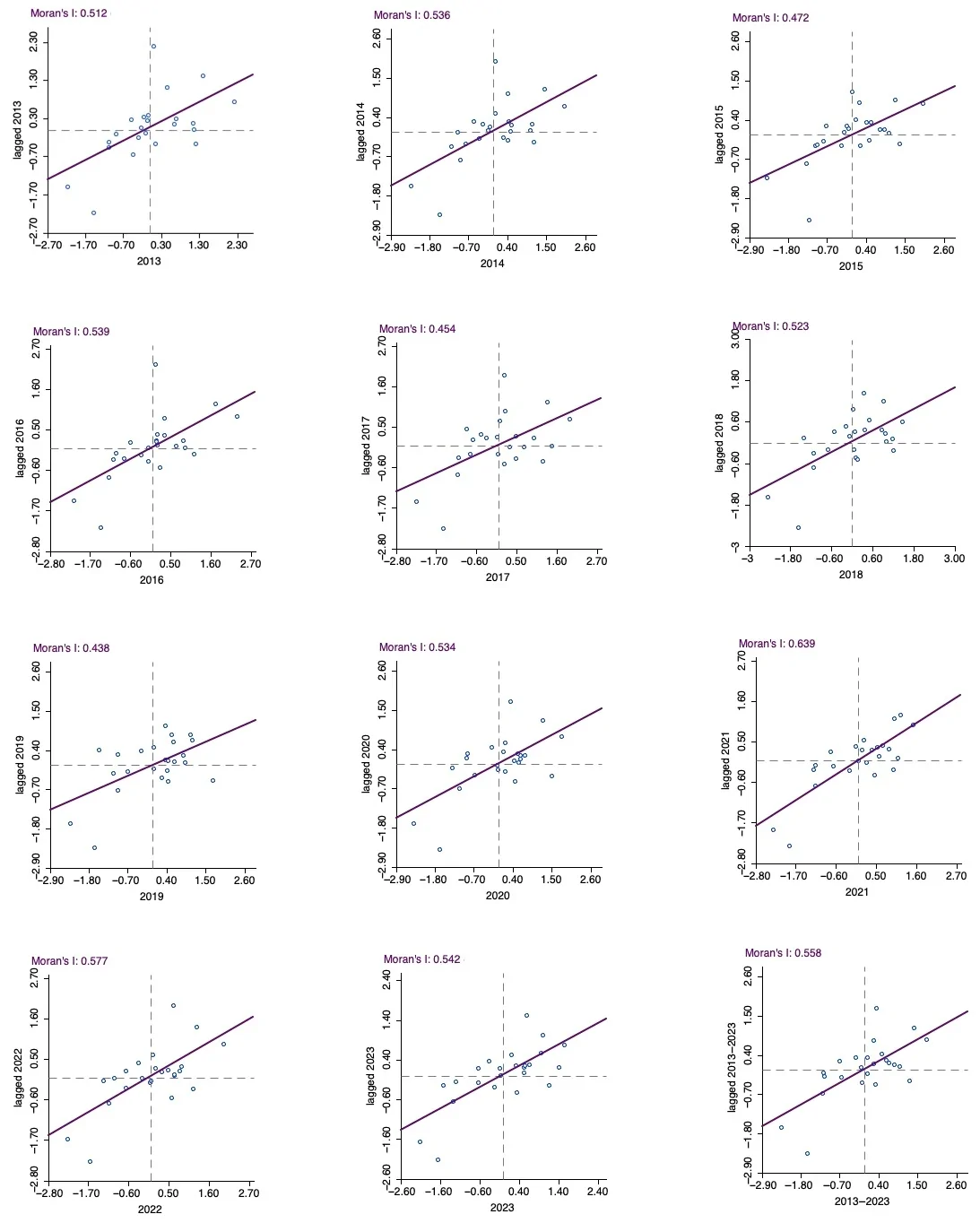
Moran’s I scatter plots illustrating the spatial autocorrelation of aminopenicillin resistance across European countries from 2013 to 2023 and for the overall study period. Blue circles represent countries; the x-axis shows standardized resistance values, the y-axis shows spatially lagged values, dashed lines indicate zero reference values, and the purple regression line represents Moran’s I.

A global Moran’s I analysis revealed that aminopenicillin resistance exhibited statistically significant positive spatial autocorrelation among European countries from 2013 to 2023. The Moran’s I coefficient ranged from 0.438 to 0.639 during the study period and was found to be positive and statistically significant in all years (*p* = 0.001 or *p* = 0.009). The lowest spatial autocorrelation was observed in 2019 (I = 0.438), and the highest in 2021 (I = 0.639). An analysis using average resistance data for the entire study period also revealed significant positive spatial autocorrelation (Moran’s I = 0.558; *p* = 0.001). The presence of positively sloped regression lines in Moran’s scatter plots for all years indicated that aminopenicillin resistance exhibited a distinct spatial clustering trend across Europe (Figure 7).

**Figure 8:**
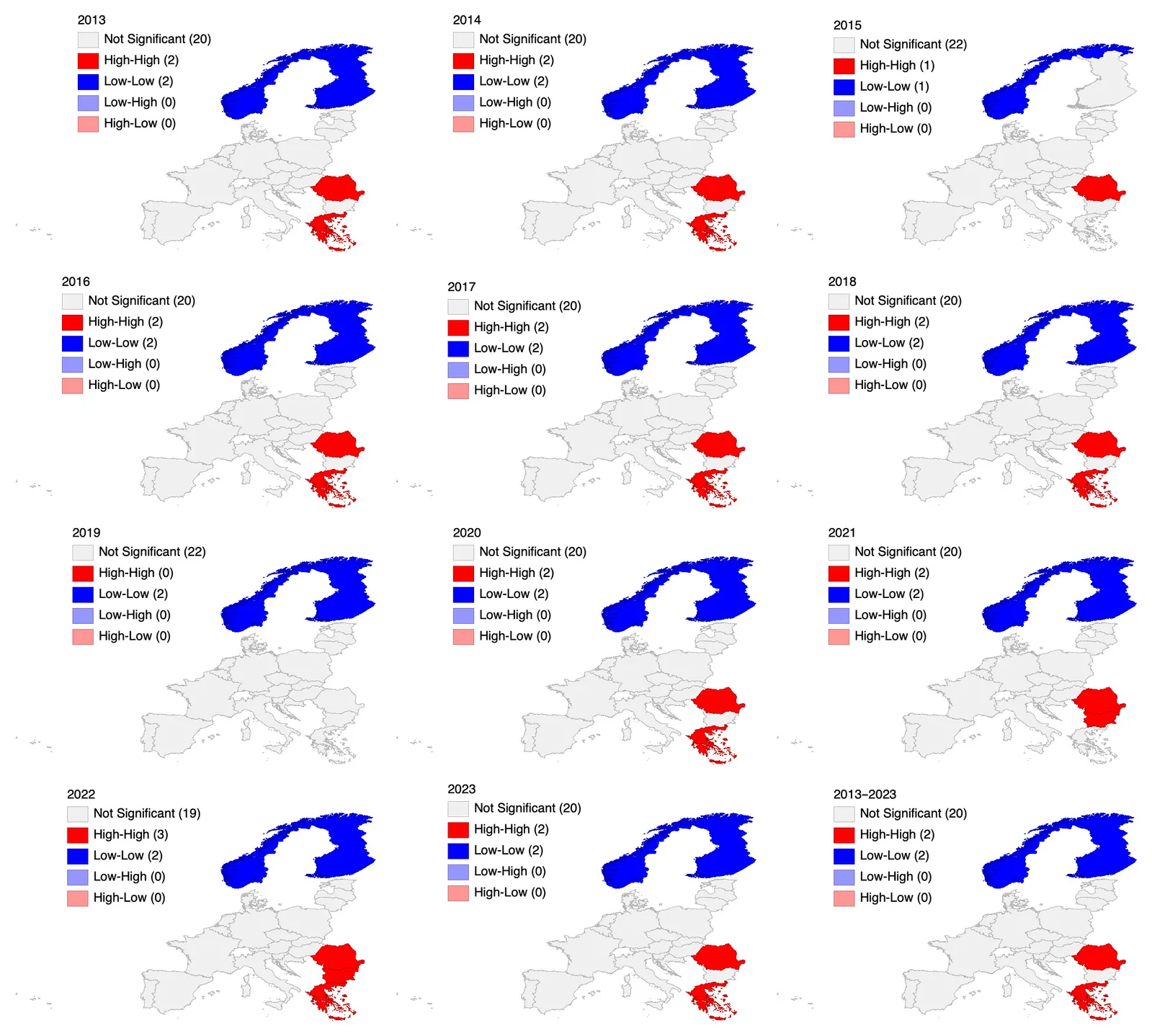
LISA cluster maps showing the spatial distribution of aminopenicillin resistance across European countries from 2013 to 2023 and for the overall study period.

The LISA analysis showed that high-resistance clusters were concentrated primarily in Southeast Europe throughout the study period. Romania, Bulgaria, and Greece formed significant High–High clusters in 2022 only. In 2013, 2014, 2016, 2017, 2018, 2020, 2023, and in the overall analysis of the study period, however, high-resistance clusters were limited to Romania and Greece. In 2015, the High–High cluster was limited to Romania alone; in 2021, it comprised Romania and Bulgaria. In 2019, no significant High–High cluster was identified. Low-resistance clusters were consistently observed throughout the entire study period in Northern European countries, Norway and Finland. These countries formed a significant Low–Low cluster in all years of the study; in 2015, the number of such clusters decreased to one, while a similar two-country pattern persisted in the other years. No Low–High or High–Low spatial outlier clusters were observed during the period under review (Figure 8).

Statistically significant positive spatial autocorrelation was detected for fluoroquinolone, aminoglycoside, and aminopenicillin resistance in all years examined. In contrast, spatial autocorrelation was not statistically significant for third-generation cephalosporin resistance in 2016 and 2017, while significant positive spatial autocorrelation was observed in all other years. In the analysis covering the entire study period, statistically significant spatial autocorrelation was detected for all four antibiotic classes, with the highest *z*-statistic observed for aminopenicillin resistance and the lowest for third-generation cephalosporin resistance. The *z*-statistics for the global Moran’s I analysis and the *p*-values from the permutation test are provided in the Supplementary Material (Table S1).

## 3. Discussion

In this study, using 11 years of balanced panel data covering 28 EU countries (27 for the aminopenicillin model, owing to insufficient Swedish data) from 2013 to 2023, the relationship between the total pesticide application intensity per cultivated area and the resistance rates to fluoroquinolones, third-generation cephalosporins, aminoglycosides, and aminopenicillins in human-derived *E. coli* isolates was examined; by including the consumption of the relevant antibiotic class as a control variable in the model and by evaluating both contemporaneous effects and effects with one-, two-, and three-year lags in separate models.

One of the most striking findings of the study is that the intensity of pesticide use showed a significant association with fluoroquinolone resistance only in the three-year lag model, whereas this association did not reach statistical significance in the same-year, one-year, and two-year lag models. This finding suggests that the effect of pesticide exposure on fluoroquinolone resistance may not be immediate, but rather follows a cumulative and delayed process. Indeed, for agricultural chemicals to exert environmental selective pressure, they must first reach a certain accumulation level in soil and water environments, and then resistant genotypes must proliferate within the population to become dominant; this two-stage process can take years [21]. In an experimental study conducted on soil microbiomes, it was shown that exposure to commonly used herbicides (glyphosate, glufosinate, and dicamba) increased the abundance of ARGs and mobile genetic elements; however, this increase did not occur immediately after herbicide application but emerged gradually over the weeks and months following application, and the selection of resistant genotypes and the spread of resistance genes among bacteria via conjugation is a process that progresses over time [22]. This finding supports the notion that the three-year delay effect observed in our study may correspond to a biologically plausible timescale.

The class-specificity of this association can also be explained mechanistically. In a study evaluating the resistance-selective potential of antibiotics at environmental concentrations, it was found that ciprofloxacin exhibited positive selection on the *intI1* gene, a marker of resistance, even at levels close to actual measured environmental concentrations; whereas macrolide antibiotics required concentrations one to two times higher than those found in the environment to exert a similar selective effect [23]. This suggests that, because fluoroquinolones are susceptible to selection even at low environmental concentrations, when considered in conjunction with additional selection pressure from pesticides, they may reach a measurable threshold more easily than other classes of antibiotics. Furthermore, in a recent review on how pesticide residues can trigger the development of AMR in agricultural ecosystems, it was suggested that structurally complex and amphiphilic pesticide compounds may act as substrates or inducers of multidrug efflux pumps, and that continuous exposure in agricultural ecosystems leads to the selection of bacterial populations with high efflux activity, thereby indirectly reducing susceptibility to antibiotics [24]. Although multidrug efflux systems such as AcrAB-TolC recognize a broad range of structurally diverse antibiotics as substrates, fluoroquinolones are among the particularly good substrates of this pump, which may partly explain why this mechanism was specifically associated with fluoroquinolone resistance in our study [25]. However, it may take longer than a single growing season for concentration-dependent cross-selection and increased efflux pump expression to translate into a detectable increase in resistance at the population level, since resistance evolution under sub- minimum inhibitory concentration (MIC) selective pressure typically proceeds through stepwise, cumulative genetic changes that require sustained longitudinal surveillance to become detectable [26,27]. This biologically supports the finding in our study that the association reached statistical significance only after a three-year lag. Furthermore, the persistence of a strong and significant association between fluoroquinolone consumption and resistance across all models indicates that this pesticide-induced delayed effect provides an additional contribution to antibiotic selection pressure rather than replacing the primary determinant, consistent with recent multivariable analyses of other environmental risk factors showing that such co-factors can contribute to AMR burden, rather than in substitution of, antibiotic consumption [28].

Although the finding that pesticide consumption did not show a statistically significant effect on third-generation cephalosporin, aminoglycoside, and aminopenicillin resistance at any delay level appears to contradict some experimental findings, it is in fact an important finding that reflects fundamental differences in the scale and design of the studies and deserves to be emphasized. Controlled experiments conducted in a laboratory setting have shown an increase in resistance to aminoglycosides, such as kanamycin and streptomycin, or to ampicillin by exposing defined bacterial strains to a single pesticide compound at known concentrations. For example, in a study examining the effects of glyphosate exposure on antibiotic resistance in soil bacteria, increases of up to approximately ninefold in the MIC of aminoglycoside antibiotics such as streptomycin and kanamycin were reported in *E. coli* strains exposed to glyphosate, and this increase was shown to be associated with mutations in the *rpsL* (30S ribosomal protein) gene [29]. Similarly, it has been reported that susceptibility to kanamycin also changes in *E. coli* and *Salmonella* strains exposed to sublethal levels of commercial herbicide formulations (based on dicamba, 2,4-D, and glyphosate), and that this change can be explained by differences in the expression of efflux pumps such as AcrAB-TolC and AcrAD-TolC [30]. However, findings in this area are not always consistent. For example, in another study conducted on *E. coli*, it was shown that glyphosate exposure did not alter the MIC against ciprofloxacin, kanamycin, and ampicillin, but only led to a temporary increase in tolerance/persistence; therefore, the observed effect may represent a temporary physiological adaptation rather than the acquisition of permanent resistance [31].

This conflicting picture demonstrates that the pesticide-antibiotic resistance relationship can vary greatly depending on the compound, strain, and experimental conditions, and this heterogeneity may become even less detectable when analyzed using aggregated national-level data from genetically diverse, multi-factorial field settings. The pesticide use indicator used in our study combines herbicides, fungicides, and insecticides into a single total value without distinguishing between specific compounds, which may limit its ability to capture compound-specific, narrow-dose-range effects. This is consistent with field-scale studies that similarly failed to detect an association between soil pesticide levels and AMR/ARG load [32]. Indeed, recent environmental risk-profiling studies identify aggregated pesticide indicators as a strong but non-specific contributor to anomalous environmental patterns associated with potential AMR risk [33]. Additionally, the countries included in the study differ substantially in agricultural systems, climate, and soil characteristics, factors recently shown to shape the spatial distribution of soil resistance genes across Europe [34], which may have increased cross-country heterogeneity and reduced the detectability of the association.

The fact that even strong and well-documented selective pressure directly linked to antibiotic use becomes apparent at the national level only over the course of years and gradually explains why a weaker and indirect selection mechanism, such as pesticide exposure, can reveal a statistically significant association at the country-year level only during specific lag periods. A study examining the relationship between antibiotic use and resistance using 11 years of national-level panel data from 26 EU countries demonstrated that even the effect of direct antibiotic use on resistance does not manifest immediately; rather, resistance prevalence continues to rise for at least four years following an increase in use, and reducing antibiotic use has only a limited effect on reducing resistance in the short term [35]. Similarly, an ecological analysis covering 30 EU countries revealed that differences in AMR across countries cannot be explained by antibiotic consumption alone; numerous competing factors, such as institutional quality, health expenditures, and socioeconomic structure, also shape resistance levels [36]. In such a system, where numerous strong determinants operate simultaneously at the national level, it is to be expected that the independent effect of a relatively indirect and aggregated environmental variable, such as pesticide use, cannot be statistically demonstrated. This situation may be particularly evident in cases of cephalosporin, aminoglycoside, and aminopenicillin resistance, where direct selective pressure associated with antibiotic consumption is high. Therefore, the findings of this study do not imply that a pesticide-resistance relationship does not exist biologically; rather, they demonstrate that an effect observed at the mechanistic level may not always be detectable at the national level using aggregated exposure data, and thus caution is warranted when directly generalizing laboratory findings to the policy level.

The fact that antibiotic consumption emerged as a much stronger and more consistent determinant than pesticide use for nearly all four resistance indicators is consistent with previous studies on AMR. Including antibiotic consumption as a control variable also reflects a methodological strength of our design, as failing to account for this major confounder is a well-recognized source of bias in observational AMR studies [37]. This is further illustrated by findings from other European-scale observational studies, which similarly underscore both the centrality of antibiotic consumption and the need to interpret its association with resistance cautiously. An observational study of 29 EU countries found moderate-to-strong correlations (r = 0.39–0.84) between national antimicrobial consumption and hospital-acquired resistance, but also notable outliers; some countries (e.g., France, Belgium, Luxembourg) showed low resistance despite high consumption, while others (e.g., Bulgaria, Hungary) showed the opposite [38]. This indicates that although antibiotic consumption is the strongest determinant of resistance, country-specific structural factors also play a role; indeed, institutional quality has been shown to affect resistance largely through pathways other than reducing consumption [39]. Our finding that antibiotic consumption remained a significant, positive determinant across all four resistance indicators, while pesticide use showed only a delayed, class-specific association, is consistent with this literature, supporting antibiotic consumption’s primary role and pesticide exposure’s more limited, complementary contribution.

The significant positive Global Moran’s I coefficients found for all four indicators confirm that AMR is not randomly distributed across Europe, with high-resistance clusters in Southeast Europe and low-resistance clusters in Northern Europe mirroring the north–south, west–east gradient reported by ECDC/WHO surveillance [40]. This gradient is unlikely to reflect a single determinant: a decade-long ecological analysis of 30 European countries found that an apparent temperature–resistance association lost significance once GDP and institutional quality were included, with antibiotic consumption and institutional quality instead emerging as the primary explanatory variables [36]. This suggests that the spatial clustering observed here likely reflects shared antibiotic use patterns, healthcare infrastructure, and regulatory policies among neighboring countries, consistent with antibiotic consumption’s role as a significant predictor across all four resistance indicators in our study.

The observation of similar levels of resistance in neighboring countries cannot be explained solely by similarities in health policies and institutional structures. Cross-border agricultural trade, the movement of people, animals, and food, as well as shared watersheds, may contribute to the spread of resistant bacteria and resistance genes between countries [41,42]. Indeed, a panel analysis at the country level has shown that antibiotic consumption in a country is associated not only with its own resistance levels but also with resistance rates in neighboring countries [35]. This finding suggests that the spatial dependence identified in our study based on the Queen neighborhood measure may reflect not only the geographical proximity of countries but also shared structural characteristics among neighboring countries and potential cross-border transmission processes. However, since the spatial analysis used did not demonstrate the direct transmission of resistance factors between countries, these potential mechanisms must be evaluated through separate epidemiological and molecular studies.

It has been demonstrated in various regions that the spatial distribution of AMR may be related to environmental conditions. For example, in a spatiotemporal analysis covering the 2014–2022 period in China, it was reported that the prevalence of carbapenem-resistant *E. coli* and *K. pneumoniae* exhibited spatial clustering associated with air pollution and climate variables [43]. Although the bacterial species, antibiotic groups, and geographic conditions examined differ from those in our study, these results indicate that the distribution of resistance may be related not only to antibiotic consumption but also to the spatial distribution of environmental and socioeconomic variables. Therefore, the combined use of panel regression and spatial autocorrelation analyses has enabled the assessment of both temporal relationships at the national level and geographic dependencies.

The potential impact of climatic and socioeconomic conditions on AMR has also been highlighted in recent global-scale studies. A comprehensive analysis evaluating climate change and socioeconomic factors together projected that improvements in sustainable development indicators could contribute significantly to reducing the global burden of AMR by 2050 [44]. In this context, the high-resistance clusters concentrated in Southeast Europe identified in our study may be associated not only with antibiotic consumption and antimicrobial use policies but also with broader structural differences such as health infrastructure, economic conditions, surveillance capacity, and environmental management. Our findings indicate that while reducing antibiotic consumption remains of fundamental importance in controlling AMR, the environmental, agricultural, and socioeconomic dimensions of the One Health approach must also be addressed collectively.

The strengths of this study include an 11-year balanced panel covering 28 European countries, standardized international databases, and the joint use of panel regression with Global Moran’s I/LISA spatial analysis to capture both temporal and spatial dependence, an approach not previously applied to pesticide–AMR relationships at this scale. Several limitations should nonetheless be noted. First, the ecological, country-year-level design precludes causal inference at the individual or strain level and carries the risk of ecological fallacy. Second, the observed association between three-year lagged pesticide use and fluoroquinolone resistance should be interpreted cautiously given the multiple comparisons involved. Pesticide use was tested separately across four resistance classes and four lag periods, of which only one reached significance, so the possibility of a Type I error cannot be excluded. While the finding’s consistency with plausible biological mechanisms lends it some support, it should not be regarded as definitive evidence of a causal or class-specific effect of pesticide use on fluoroquinolone resistance, but rather as an exploratory, hypothesis-generating finding that warrants confirmation in future research. Third, the aggregated pesticide indicator does not distinguish between compounds or directly measure environmental residue concentrations, on which resistance selection thresholds actually depend [23]. Fourth, veterinary antibiotic use, livestock density, and fertilizer application were not included and remain potential confounders. Finally, the three-year lag cannot be definitively attributed to a direct pesticide-driven mechanism rather than an unmeasured, co-evolving agricultural practice.

Future studies should incorporate compound-specific residue measurements alongside environmental ARG monitoring [45], apply spatial panel models such as the spatial Durbin model to separate within-country from cross-border effects [43], and include veterinary antimicrobial use and livestock intensity as covariates. Interdisciplinary, long-term monitoring remains essential to disentangle these multifaceted relationships [46].

## 4. Materials and Methods

### 4.1. Data Sources and Data Collection

This study was designed as a country-level longitudinal ecological panel data study covering the period 2013–2023. The study includes a total of 28 European countries: Austria, Belgium, Bulgaria, Croatia, Cyprus, the Czech Republic, Denmark, Estonia, Finland, France, Germany, Greece, Hungary, Ireland, Italy, Latvia, Lithuania, Luxembourg, Malta, the Netherlands, Norway, Poland, Portugal, Romania, Slovakia, Slovenia, Spain, and Sweden. All data on AMR, antibiotic consumption, and pesticide use in the study were obtained from international open-access databases on 25 June 2026.

AMR data were obtained from the European Antimicrobial Resistance Surveillance Network (EARS-Net) database, coordinated by the ECDC “https://atlas.ecdc.europa.eu/public/index.aspx (accessed on 25 June 2026)”. This study utilized resistance data for *E. coli* isolates obtained from humans against fluoroquinolones, aminopenicillins, aminoglycosides, and third-generation cephalosporins. The data were filtered by country, year, bacterial species, and antimicrobial class, and the percentage of resistant isolates among all tested isolates was used as the dependent variable. Since data for the 2013–2023 period were available for all 28 countries in the fluoroquinolone, aminoglycoside, and third-generation cephalosporin resistance analyses, balanced panel datasets consisting of 308 country-year observations were created for each resistance indicator. Since aminopenicillin resistance data for Sweden were available only for 2013 and 2015, and sufficient data were not available for the remainder of the study period, Sweden was entirely excluded from the aminopenicillin analysis. Consequently, the aminopenicillin model was run using a separate balanced panel dataset consisting of 297 country-year observations from 27 countries.

Antibiotic consumption data were obtained from the interactive database of the European Surveillance of Antimicrobial Consumption Network (ESAC-Net), coordinated by the ECDC “https://qap.ecdc.europa.eu/public/extensions/AMC2_Dashboard/AMC2_Dashboard.html#who-aware-tab (accessed on 25 June 2026)”. Under the main group J01, which covers antibacterials for systemic use, the antimicrobial consumption group associated with each resistance indicator was selected separately. Accordingly, consumption data from the ATC groups J01DD for third-generation cephalosporin resistance, J01C for aminopenicillin resistance, J01MA for fluoroquinolone resistance, and J01G for aminoglycoside resistance were used. For each country and year, the “Total care (community and hospital sector)” option, which covers both the community and hospital sectors, was used, and consumption amounts were recorded in metric tons per year, representing the annual total usage. Antibiotic consumption was included in the relevant panel regression model as a key control variable in the assessment of the relationship between pesticide use and *E. coli* resistance rates.

Pesticide use data were obtained from the pesticide use section of the Food and Agriculture Organization Corporate Statistical Database (FAOSTAT) provided by the Food and Agriculture Organization of the United Nations (FAO) “https://www.fao.org/faostat/en/#data/RP (accessed on 25 June 2026)”. Total pesticide use was defined using the “use per area of cropland” measure under the “pesticides (total)” indicator and expressed in kilograms per hectare (kg/ha) of cropland. The dataset included annual observations for the 28 countries covered by the study during the 2013–2023 period and consisted of a total of 308 country-year observations. Pesticide use intensity was used as the primary independent variable in the panel regression models.

All datasets were downloaded from the relevant databases in CSV format; where necessary for data verification, editing, and merging, the files were converted to “.xlsx” format. AMR data, consumption data for the relevant antibiotic groups, and pesticide use data were matched using country and year variables as common identifiers. Following the merging process, the country-year scope, variable definitions, and units of measurement were verified, and a separate analysis dataset was created for each resistance indicator. Consequently, analyses of fluoroquinolones, aminoglycosides, and third-generation cephalosporins were conducted using balanced panel data structures covering 28 countries, while the analysis of aminopenicillins was conducted using a balanced panel data structure covering 27 countries.

### 4.2. Panel Data Regression Analysis

To assess the relationship between pesticide use and AMR, a panel data analysis was conducted using annual observations from 28 European countries between 2013 and 2023. In the analyses, the resistance rates for fluoroquinolones, third-generation cephalosporins, aminoglycosides, and aminopenicillins, each transformed using the natural logarithm, were considered separately as dependent variables. For each resistance model, annual antibiotic consumption data for the relevant antibiotic class were included in the model as a control variable.

Four separate panel regression models were constructed to examine the simultaneous and lagged effects of pesticide consumption on AMR. In the first model, pesticide consumption for the same year was used as the independent variable; in the second model, pesticide consumption with a one-year lag (Lag 1) was used; in the third model, pesticide consumption with a two-year lag (Lag 2) was used; and in the fourth model, pesticide consumption with a three-year lag (Lag 3) was used. The lagged pesticide variables were generated in Stata software (StataCorp LLC, College Station, TX, USA; version 12) after the panel data structure was defined.

The panel data regression model was defined using the following general equation:

$$\ln\left(AMR_{it}\right)=\beta_{0}+\beta_{1}Pesticide_{i,t-k}+\beta_{2}AntibioticUse_{it}+u_{i}+\varepsilon_{it}$$

Here,

- $\ln\left(AMR_{it}\right)$: the natural logarithm of the antimicrobial resistance rate in country *i* in year *t*;
- $Pesticide_{i,t-k}$: pesticide consumption in the same year (*k* = 0) or with a lag (*k* = 1, 2, 3);
- $AntibioticUse_{it}$: the annual consumption volume for the relevant antibiotic group;
- $\beta_{0}$: the constant term;
- $\beta_{1}$ and $\beta_{2}$: the regression coefficients;
- $u_{i}$: the country-specific, time-invariant, unobservable random effect;
- $\varepsilon_{it}$: the error term.

In the panel data structure, the country variable was defined as the panel variable and the year variable as the time variable. First, fixed-effects and random-effects models were estimated. The Hausman specification test was used to determine the appropriate model. Since the Hausman test revealed no statistically significant difference between the fixed-effects and random-effects models (χ^2^ = 1.31; *p* = 0.519), the random-effects generalized least squares (GLS) panel regression model was used in the analyses. Regression results were reported as the regression coefficient (*β*), the 95% confidence interval (95% CI), and the *p*-value. A *p*-value of less than 0.05 was considered statistically significant.

### 4.3. Spatial Autocorrelation Analysis

A Global Moran’s I analysis was conducted to assess the spatial distribution of AMR data across 28 European countries. The analysis utilized a first-order Queen’s contiguity weighting matrix. In these analyses, although the dataset included 28 European countries, Ireland, Cyprus, and Malta had no contiguous neighbours under the Queen contiguity spatial weights matrix and were therefore treated as spatial isolates and excluded from the analyses. The Moran’s I coefficient was calculated using the following equation:

$$I=\frac{N}{S_{0}}\frac{\sum_{i=1}^{N}\sum_{j=1}^{N}w_{ij}(x_{i}-\overset{ˉ}{x})(x_{j}-\overset{ˉ}{x})}{\sum_{i=1}^{N}(x_{i}-\overset{ˉ}{x}{)}^{2}}$$

Here,

- N: the number of countries analyzed;
- $x_{i}$: the antimicrobial resistance value of country *i*;
- $\overset{ˉ}{x}$: the average resistance value of all countries;
- $w_{ij}$: the spatial weight coefficient between countries *i* and *j*;
- $S_{0}=\sum_{i}\sum_{j}w_{ij}$: represents the total spatial weight value.

To determine the location of clusters for resistance groups where the Global Moran’s I analysis was found to be statistically significant, the Local Indicators of Spatial Association (LISA) analysis was applied. In the LISA analysis, countries were classified into four spatial relationship categories: High–High, Low–Low, High–Low, and Low–High. The statistical significance of local clusters was assessed at the *p* < 0.05 level using 999 permutations. All panel data analyses were performed using Stata software (version 12; StataCorp LLC, College Station, TX, USA), while spatial analyses were conducted using GeoDa software (version 1.22; Center for Spatial Data Science, University of Chicago, Chicago, IL, USA). QGIS (version 3.44; QGIS Development Team, QGIS Association, Laax, Switzerland) was used to prepare the thematic maps.

## 5. Conclusions

In conclusion, this study is one of a limited number of country-level ecological studies that assess the relationship between pesticide use and AMR in *E. coli* in terms of time lags and together with the spatial patterns of AMR, based on 11 years of data covering 28 European countries. The findings show that, among the four resistance indicators examined, only fluoroquinolone resistance was significantly and positively associated with pesticide use with a three-year lag. In contrast, no significant association was found between pesticide use and third-generation cephalosporin, aminoglycoside, or aminopenicillin resistance at any of the lag periods evaluated, and this single significant finding should be interpreted cautiously in light of the multiple comparisons performed. Consumption of the relevant antibiotic classes, however, showed consistent and positive relationships with resistance levels in the majority of the models examined. These results suggest that antibiotic consumption remains a key determinant of AMR, while the potential contribution of pesticide use may be more limited, delayed, and specific to the type of resistance.

The significant and positive spatial autocorrelation observed across all four resistance indicators, along with clusters of high resistance concentrated in Southeast Europe and clusters of low resistance concentrated in Northern Europe, demonstrates that AMR is not randomly distributed across Europe but exhibits a distinct regional pattern. This spatial pattern indicates that resistance management cannot be limited to national interventions alone; differences in antibiotic usage patterns, agricultural and commercial mobility, health infrastructure, and regulatory capacity among neighboring countries must also be taken into account. While our findings support the central importance of antimicrobial stewardship programs in combating resistance, they also indicate that the potential delayed and cumulative effects of agricultural pesticide use must be assessed in greater detail through composite exposure data, monitoring of ARGs in the environment, and long-term regional studies.

## Figures and Tables

**Table 1:** Estimated effects of current and lagged pesticide exposure on antimicrobial resistance from multivariable panel regression models, adjusted for antibiotic consumption.

Outcome	Pesticide Exposure	*β* (Pesticide)	95% CI	*p*	*β* (Antibiotic Consumption)	*p*
Fluoroquinolone resistance	Current year	0.000068	−0.000054 to 0.000190	0.275	0.0119	<0.001
	Lag 1 year	0.000063	−0.000072 to 0.000197	0.359	0.0128	<0.001
	Lag 2 years	0.000115	−0.000010 to 0.000241	0.072	0.0133	<0.001
	Lag 3 years	0.000181	0.000047 to 0.000315	0.008	0.0143	<0.001
Third-generation cephalosporin resistance	Current year	−0.000090	−0.000232 to 0.000052	0.215	0.0196	0.001
	Lag 1 year	0.000077	−0.000068 to 0.000222	0.3	0.0238	<0.001
	Lag 2 years	0.000031	−0.000092 to 0.000154	0.617	0.0238	<0.001
	Lag 3 years	−0.000055	−0.000183 to 0.000073	0.401	0.0216	<0.001
Aminoglycoside resistance	Current year	0.000073	−0.000083 to 0.000228	0.36	0.1517	0.009
	Lag 1 year	0.000068	−0.000094 to 0.000230	0.411	0.1519	0.006
	Lag 2 years	0.000038	−0.000120 to 0.000196	0.638	0.1633	0.003
	Lag 3 years	−0.000063	−0.000227 to 0.000100	0.448	0.1133	0.052
Aminopenicillin resistance	Current year	0.000007	−0.000044 to 0.000057	0.799	0.000513	0.001
	Lag 1 year	0.000002	−0.000054 to 0.000058	0.944	0.000521	0.001
	Lag 2 years	0.000011	−0.000045 to 0.000067	0.694	0.000524	0.001
	Lag 3 years	0.000012	−0.000048 to 0.000071	0.702	0.000468	0.004

## Data Availability

The data presented in this study are available upon request from the corresponding author due to privacy restrictions.

## Supplementary Materials

The following supporting information can be downloaded at https://www.mdpi.com/article/10.3390/microorganisms14092018/s1. Table S1: Global Moran’s I statistics for the spatial autocorrelation of fluoroquinolone, third-generation cephalosporin, aminoglycoside, and aminopenicillin resistance across European countries between 2013 and 2023.

## Abbreviations

The following abbreviations are used in this manuscript:

AMR	Antimicrobial resistance
ARGs	Antibiotic resistance genes
EARS-Net	European Antimicrobial Resistance Surveillance Network
ECDC	European Centre for Disease Prevention and Control
ESAC-Net	European Surveillance of Antimicrobial Consumption Network
EU	European Union
FAO	Food and Agriculture Organization of the United Nations
FAOSTAT	Food and Agriculture Organization Corporate Statistical Database
LISA	Local spatial autocorrelation
MIC	Minimum inhibitory concentration
WHO	World Health Organization
