# Supplementary material for: Agricultural Pesticide Exposure and Antimicrobial Resistance in Escherichia coli Across 28 European Countries: A Panel and Spatial Data Analysis
Source: Microorganisms. 2026 Sep 11;14(9):2018. doi: 10.3390/microorganisms14092018 (PMC13609689; doi:10.3390/microorganisms14092018)
Supplement: Supplementary file 1 [file microorganisms-14-02018-s001.zip › Table S1.pdf]

Table S1. Global Moran's I statistics for the spatial autocorrelation of fluoroquinolone, third-generation cephalosporin, aminoglycoside, and aminopenicillin resistance across European countries between 2013 and 2023.

	**Fluoroquinolone**		**Third-generation cephalosporin**		**Aminoglycoside**		**Aminopenicillin**	
**Year**	***z*-value**	***p*-value**	***z*-value**	***p*-value**	***z*-value**	***p*-value**	***z*-value**	***p*-value**
2013	2.318	0.009	2.379	0.015	2.838	0.01	3.739	0.001
2014	2.427	0.006	3.037	0.005	3.046	0.004	3.874	0.001
2015	1.962	0.022	2.685	0.008	2.476	0.011	3.619	0.003
2016	2.106	0.019	1.730	0.053	2.861	0.004	3.803	0.001
2017	1.799	0.032	1.735	0.052	2.611	0.006	3.664	0.001
2018	2.212	0.012	2.046	0.025	2.849	0.004	3.933	0.001
2019	2.167	0.012	1.983	0.034	1.920	0.025	3.631	0.001
2020	2.281	0.007	2.369	0.008	2.806	0.003	3.917	0.001
2021	2.493	0.006	2.894	0.003	3.189	0.001	4.285	0.001
2022	2.904	0.002	3.048	0.002	3.469	0.001	4.022	0.001
2023	2.556	0.008	2.643	0.004	3.098	0.003	3.893	0.001
**2013–2023**	**2.394**	**0.008**	**2.567**	**0.009**	**3.175**	**0.003**	**3.980**	**0.001**
